# Exercise combined with Acceptance and Commitment Therapy (ExACT) compared to a supervised exercise programme for adults with chronic pain: study protocol for a randomised controlled trial

**DOI:** 10.1186/s13063-018-2543-5

**Published:** 2018-03-22

**Authors:** Máire-Bríd Casey, Keith Smart, Ricardo Segurado, Conor Hearty, Hari Gopal, Damien Lowry, Dearbhail Flanagan, Lance McCracken, Catherine Doody

**Affiliations:** 1 School of Public Health, Physiotherapy and Sports Science, Health Sciences Building, University College Dublin, Belfield, Dublin 4, Ireland; 2 Physiotherapy Department, St Vincents University Hospital, Elm Park, Dublin 4, Ireland; 3 Department of Pain Medicine, Mater Misericordiae University Hospital, Eccles St, Dublin 7, Ireland; 4 Psychology Department, Mater Misericordiae University Hospital, Eccles St, Dublin 7, Ireland; 5 Physiotherapy Department, Mater Misericordiae University Hospital, Eccles St, Dublin 7, Ireland; 6 Psychology Department, Institute of Psychiatry, Psychology and Neuroscience, Kings College London, 5th Floor Bermondsey Wing, Guy’s Campus, London, SE1 9RT United Kingdom

**Keywords:** Chronic pain, Exercise, Acceptance and commitment therapy, Multidisciplinary pain programme, Physical therapy, Physiotherapy, Psychological therapy

## Abstract

**Background:**

Acceptance and Commitment Therapy (ACT) is a form of cognitive behavioural therapy, which may be beneficial for people with chronic pain. The approach aims to enhance daily functioning through increased psychological flexibility. Whilst the therapeutic model behind ACT appears well suited to chronic pain, there is a need for further research to test its effectiveness in clinical practice, particularly with regards to combining ACT with physical exercise.

**Methods/design:**

This prospective, two-armed, parallel-group, single-centre randomised controlled trial (RCT) will assess the effectiveness of a combined Exercise and ACT programme, in comparison to supervised exercise for chronic pain. One hundred and sixty patients, aged 18 years and over, who have been diagnosed with a chronic pain condition by a physician will be recruited to the trial. Participants will be individually randomised to one of two 8-week, group interventions. The combined group will take part in weekly psychology sessions based on the ACT approach, in addition to supervised exercise classes led by a physiotherapist. The control group will attend weekly supervised exercise classes but will not take part in an ACT programme. The primary outcome will be pain interference at 12-week follow-up, measured using the Brief Pain Inventory-Interference Scale. Secondary outcomes will include self-reported pain severity, self-perception of change, patient satisfaction, quality of life, depression, anxiety and healthcare utilisation. Treatment process measures will include self-efficacy, pain catastrophising, fear avoidance, pain acceptance and committed action. Physical activity will be measured using Fitbit Zip^TM^ activity trackers. Both groups will be followed up post intervention and again after 12 weeks. Estimates of treatment effects at follow-up will be based on an intention-to-treat framework, implemented using a linear mixed-effects model. Individual and focus group qualitative interviews will be undertaken with a purposeful sample of participants to explore patient experiences of both treatments.

**Discussion:**

To our knowledge, this will be the first RCT to examine whether combining exercise with ACT produces greater benefit for patients with chronic pain, compared to a standalone supervised exercise programme.

**Trial registration:**

www.ClinicalTrials.gov, ID: NCT03050528. Registered on 13 February 2017.

**Electronic supplementary material:**

The online version of this article (10.1186/s13063-018-2543-5) contains supplementary material, which is available to authorized users.

## Background

Chronic pain is a major health problem, reported to affect 19% of adult Europeans [1] and up to 35.5% of Irish adults [2]. The economic burden is significant, with a recent survey in Ireland estimating the total cost of treating chronic pain at €5.34 billion per year [3]. This survey of 1204 Irish people also reported that health-related quality of life was significantly lower in people with chronic pain compared to people without pain, and that depression was significantly higher [2].

Chronic pain has been defined as an unpleasant sensory or emotional experience, associated with actual or potential tissue damage, which persists for over 3 months’ duration [4]. The multidimensional nature of chronic pain presents significant challenges for patients and healthcare professionals. There is a plethora of treatment options available but the effects of these interventions on pain and disability are modest and improvements are typically short term [5, 6]. Traditional biomedical interventions, such as surgery and spinal injections, have not been shown to be superior to conservative treatments for chronic pain and they carry greater risks [7, 8]. Exercise interventions and psychological therapies, such as cognitive behavioural therapy (CBT), are examples of conservative treatments that are known to be effective for patients with chronic pain [5, 6, 9–12]. These interventions can be provided individually or they can be effectively combined in the form of a multidisciplinary biopsychosocial rehabilitation programme [13].

Physical activity is an important outcome to target with chronic pain interventions, as in addition to the physical limitations imposed by pain, there are strong associations with cardio-metabolic and respiratory conditions [14]. Exercise, including aerobic, strengthening and aquatic exercise has been shown to reduce pain and improve physical function and quality of life [15–17], but the quality of the evidence is low and further studies with larger samples are required [12]. No particular type of exercise has been shown to be superior to another [5, 18] and research suggests that group-based physiotherapy interventions incorporating exercise are just as effective for pain and disability as individual treatment [19]. Patient adherence to treatment should be promoted by providing individualised exercises within supervised programmes, and supplementing with home exercises [20].

There is a large evidence base related to psychological treatments for chronic pain, with CBT being the dominant intervention. A Cochrane review concluded that CBT has small to moderate effects on pain, disability, mood and catastrophising [6]. The authors noted that whilst there have been improvements in the methodological quality of studies in recent years, there has been no change in the overall effects of the interventions and they recommend against further randomised controlled trials (RCTs) examining the efficacy of CBT.

Acceptance and Commitment Therapy (ACT) is a psychological therapy that encourages participants to change their relationship with their thoughts and physical sensations through mechanisms of acceptance, mindfulness and value-based action [21]. Systematic reviews of RCTs featuring ACT for adults with chronic pain have reported that ACT is effective for enhancing general function and decreasing distress, compared to inactive treatment comparisons [22, 23]. One RCT included in these reviews compared ACT with CBT and found no significant differences in improvement between the two treatments; however, greater levels of satisfaction were reported by the ACT participants [24]. Another RCT reported equivalent reductions in pain and disability with ACT, when compared with applied relaxation for chronic pain [25]. Whilst there is growing evidence to support the effectiveness of ACT, it has been acknowledged that there are currently only a small number of high-quality studies and further RCTs have been recommended, in particular with active treatment comparisons [9, 22].

There are currently no RCTs that have examined the effectiveness of exercise combined with ACT for chronic pain. Furthermore, in the RCTs published to date, ACT as a standalone therapy has not been shown to be effective in enhancing physical activity [26, 27]. When CBT and ACT were compared for chronic pain, no significant improvement in physical activity was observed for either psychological approach [27] and the authors suggest that tailored interventions, with greater emphasis on exercise, may complement psychological treatment for chronic pain. To our knowledge, this will be the first RCT to assess the effectiveness of a combined Exercise and ACT intervention for chronic pain.

### Research objectives

The primary objective is to determine whether a combined Exercise and ACT group-based intervention is effective for reducing pain interference at 12-week follow-up, in patients with chronic pain, compared to a physiotherapy-led supervised exercise programme.

#### Secondary objectives

1. To investigate whether exercise combined with ACT has a positive impact on study participants compared to a supervised exercise programme, with regard to the self-reported secondary outcomes: pain severity, self-perception of change, patient satisfaction, quality of life, depression, anxiety and healthcare costs, and treatment process outcomes: self-efficacy, pain catastrophising, fear avoidance, pain acceptance and committed action following treatment and at 12-week follow-up
2. To examine whether exercise combined with ACT has a significant effect on objective physical activity measures (step count, distance travelled and active minutes) post treatment, compared to a supervised exercise programme
3. To explore the experiences of a purposeful sample of participants of both interventions with embedded qualitative interviews

## Methods/design

### Study design

The ExACT trial is a two-armed, single-centre, parallel-group, randomised controlled superiority trial.

### Setting

Participants will be recruited from a consultant-led pain clinic and musculoskeletal out-patient clinics within a secondary care setting of a large academic teaching hospital in Dublin, Ireland. Treatments will take place in the pain clinic and in the physiotherapy department of the hospital.

### Participants

**Fig. 1:**
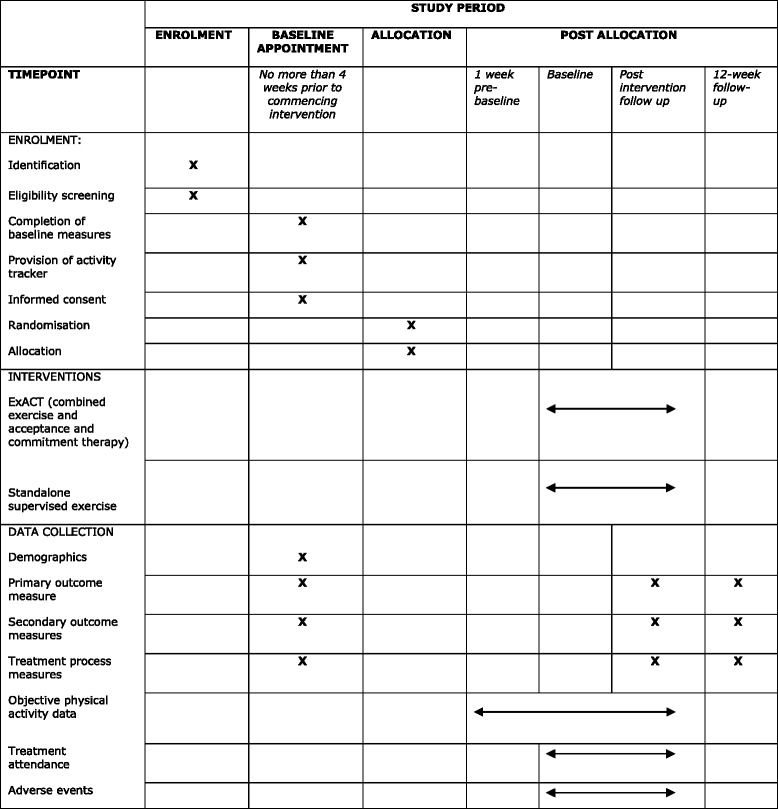
Standard Protocol Items: Recommendations for Interventional Trials (SPIRIT) Diagram

A total of 160 participants will be randomised to the combined Exercise and ACT or supervised exercise groups over a 20-month period. Adults (aged 18 years and older) with any type of chronic pain condition, other than cancer pain, (diagnosed by a physician), which is persisting for over 12 weeks’ duration and who report a score of ≥ 2 on the Brief Pain Inventory-Interference Scale (BPIIS) are eligible for inclusion in the study. Participants must also be able to provide informed consent and communicate effectively in the English language. Figure 1 shows the Standard Protocol Items: Recommendations for Interventional Trials (SPIRIT) Diagram for the trial.

Exclusion criteria are as follows: need for further diagnostic evaluation (determined by a physician), presence of major medical or psychiatric disorder that would impede ability to participate with treatment), presence of active cancer or cancer-related pain, unstable inflammatory condition (e.g. rheumatoid arthritis, gout), presence of substance misuse, surgical or interventional procedure (e.g. spinal cord stimulator, rhizotomy, epidural or intra-articular injection) within the last 3 months, concurrent participation (or participation within the last 3 months) in a supervised exercise programme or a course of psychological or physiotherapy treatment, previous participation in any multidisciplinary pain management programme or presence of any contraindication to participation in a gym or pool-based exercise programme (e.g. shortness of breath at rest, unstable diabetes or epilepsy, recent myocardial infarction, stroke, pulmonary embolism, asthma attack, weight > 125 kg (19.5 stone) or waist circumference > 50 in. (restriction due to hydrotherapy evacuation equipment).

### Participant identification, recruitment and consent

Adults, who attend hospital out-patient clinics for treatment of chronic pain will be screened for study eligibility by a physician. The number of patients who undergo screening will be recorded in order to quantify the number of patients who are deemed eligible or ineligible for the study and how many patients decline to participate. The reasons for ineligibility will be recorded. Those who meet the eligibility criteria will be informed about the study by their physician and written information in the form of a Patient Information Leaflet will be provided. Patients who express interest in participating in the study will be contacted by telephone by the lead researcher (MBC). Any questions will be clarified on the telephone and patients who remain interested will be invited to attend an individual face-to-face appointment with the lead researcher in the hospital pain clinic. Baseline outcome measures will be sent in advance by post and the patients will be asked to bring the completed questionnaires with them on the day of their appointment.

Informed consent will be obtained in writing by the lead researcher, prior to participation in the study. Patients will be informed that they are under no obligation to participate and they may withdraw their consent at any time without need for explanation. Where possible the reasons for withdrawal from the trial will be recorded. Patients who do not wish to take part in the study will continue to have treatment as usual. A sample size target of 160 participants over a 20-month period has been set. Recruitment will be monitored throughout the trial and if expected rates of recruitment are not being achieved, additional patients may be recruited via the physiotherapy waiting list or paper triage of referral letters and patient databases, performed by physicians, with subsequent eligibility screening by the lead researcher.

### Interventions

The study interventions are described below and are written with reference to the TIDieR guidelines for better reporting of interventions [28].

#### Combined Exercise and Acceptance and Commitment Therapy (ExACT)

ExACT is a multidisciplinary pain programme combining exercise and psychological therapy. It is a face-to-face, group-based treatment, with up to ten individuals per group. Participants will attend a total of eight sessions, once per week, with each session lasting 3.5 h. Each day will begin with a 2-h psychology session held in the hospital pain clinic. The sessions will follow the psychological approach Acceptance and Commitment Therapy (ACT) and are designed to promote psychological flexibility through methods that encourage openness, awareness and engagement. The overall aim is to reduce pain-avoidant behaviours, in the service of living a rich and meaningful life [21]. Group discussions, experiential exercises and mindfulness practice (see Table 1) will be led by a psychologist who has been trained in ACT and is experienced in treating chronic pain. The content of the sessions will be adapted from an ACT treatment manual used in a recently published study [29] and available to members of the Association for Contextual Behavioral Science (ACBS) from the website http://www.contextualscience.org/better_living_with_illness. Written supplementary material will be provided each week and participants will be encouraged to spend time reviewing the material at home.

**Table 1:** Summary of the content of the Acceptance and Commitment Therapy (ACT) component of the combined intervention

Session	Content
1	Introductions and basic foundations of treatment, present the goal of ACT – shifting focus from pursuit of symptom reduction to improving function
2	Review of previous treatment history – creative hopelessness exercise including primary and secondary suffering Introduce openness as a skill area – acceptance as an alternative to avoidance
3	Recap of acceptance and continued focus on enhancing openness. Introduce process of defusion. Passenger on the bus experiential exercise
4	Focus on engagement: values awareness and assessment Experiential values exercise
5	Further values clarification work Committing to action that improves and enriches one’s life
6	Focus on awareness – contact with the present moment, perspective taking and self-awareness as distinct from fusion with thought content and perception of self
7	Treatment review Walking mindfulness exercise
8	Wrap up and conclusions Relapses and set-backs: preparation not prevention

Following a break for lunch, participants will attend a 1.5-h supervised exercise class in the physiotherapy department of the hospital. The classes will be delivered by a physiotherapist and will feature two components: (1) education/advice and (2) exercise (see Table 2). The education/advice sessions will be interactive and will take the form of group discussions of approximately 30-min duration, covering topics such as goal-setting, understanding pain, physical activity and pacing. The physiotherapist will answer questions and facilitate discussion related to relevant issues that the participants bring to the group. The exercise sessions will take place in either a pool or a gym setting (four sessions of each). The aquatic sessions will include a warm up, gentle aerobic exercise, buoyancy-assisted and resisted movements, and informal ball games. The gym programme will feature a combination of gentle aerobic exercise, stretches and strengthening exercises. The specific exercises will be chosen by the physiotherapist and examples will include, but will not be limited to: cycling on a static bike and treadmill walking, pulleys, sit to stands, step-ups, wall squats, wall press-ups, seated flexion, trunk rotation in standing, knee rolling/trunk rotation on plinth, bridging, knees to chest. The exercise programmes will be individualised, based on each participant’s personal goals and a written exercise programme, compiled by the physiotherapist will be provided. The programmes will be progressed and modified for each individual as deemed appropriate by the physiotherapist. Participants will be encouraged to carry out their exercises at home or in their local pool. Throughout the aquatic and gym exercise sessions, there will be an emphasis on reducing threat and fear of movement. The physiotherapist will encourage improved physical activities in a manner that gradually increases physical function and enhances enjoyment of physical activity. The physiotherapist leading the supervised exercise programmes will have over 7 years of experience treating patients with chronic pain, including the delivery of group exercise programmes to patients with similar conditions to the trial participants. The physiotherapist will not have had formal training in ACT, ensuring that only the participants of the combined group will be exposed to this form of psychological therapy.

**Table 2:** Summary of the content of the supervised exercise component of both interventions

Session	Education/advice (30 min)	Exercise (1 h)
1	Introduction to exercise Pool orientation Induction to gym programme Demonstration of gym exercises	Gym exercise: Gentle warm up – walking and stretches Brief gym circuit Cool down
2	Group discussion on goal setting Provision of individual home exercise programme HEP created by physiotherapist based on patient’s individual goals	Hydrotherapy Warm up Gentle aerobic and buoyancy assisted and resisted exercises
3	Understanding pain Group will be shown the YouTube video ‘understanding pain in 5 minutes’ followed by a group question/answer session	Hydrotherapy Warm up Aerobic, strengthening exercises and informal pool games
4	Group discussion about physical activity, introduction to pacing and principles of graded exposure Time to answer any questions from participants	Hydrotherapy Continuation of above and gentle progression of exercises
5	Group discussion on pacing and graded exposure including potential challenges that may be arising regarding putting principles into practice	Hydrotherapy Continuation of above and gentle progression of exercises
6	Continued group discussion on progress and problem solving	Gym session – participants are free to perform exercises from their individualised exercise programme or other exercises of their own choosing under the guidance of the physiotherapist
7	Group discussion on progress and problem solving. Introduce topic of maintaining behaviour change	Gym session Continuation of above and progression of exercise under guidance of physiotherapist
8	Wrap-up session including preparation for maintaining an active lifestyle and managing setbacks	Gym session Continuation of above and progression of exercise under guidance of physiotherapist

#### Standalone supervised exercise

The standalone, supervised exercise programme will also consist of eight face-to-face, 1.5-h sessions delivered by a physiotherapist, on a weekly basis, to groups of up to ten participants. The intervention will mirror the supervised exercise component of the combined treatment as described above.

### Treatment adherence and other interventions

Attendance at both interventions will be recorded by the treating clinicians. Participants will be encouraged to inform the administrative staff in the pain clinic by telephone or email if they are unable to attend and, where possible, the reasons for absence will be recorded. Attendance rates will be reported with the trial results.

All study participants will continue to attend routine medical appointments with their general practitioner or hospital consultants for the duration of the trial. These appointments will be recorded and reported. Other than the trial interventions, participants will be asked to refrain from additional treatment provided by allied health practitioners, such as psychologists, counsellors, physiotherapists or complementary therapists, during the 8-week treatment period. Any medication changes and the administration of any additional interventions during the course of the trial will be recorded and reported, and reasons for same will be documented. Patients will not be denied any treatments that a physician deems necessary to administer urgently.

### Treatment fidelity

Assessment of treatment fidelity is an important component in ensuring transparency in clinical research and increasing confidence that the intervention is delivered as described [30]. Treatment fidelity of the ACT intervention in this trial will be assessed by a health psychologist (NL), who is highly experienced in the delivery of group psychological interventions for chronic pain using ACT. All eight ACT sessions, from one treatment group will be audio-recorded and sent to NL for review. An ACT treatment fidelity tool that has been modified for chronic pain will be used to evaluate the intervention [31]. The psychologist delivering the ACT intervention will complete written notes, detailing the content covered within each session and a brief note outlining any relevant observations. These will be reviewed alongside the audio-recordings.

The treating physiotherapist will also complete checklists after each exercise session and will record any additional relevant details. Treatment fidelity of the physiotherapy components of the trial will be assessed by another member of the project team (KS), a practising clinical specialist physiotherapist, who will review the checklists and notes at monthly intervals.

### Randomisation

Randomisation will take place after baseline measures have been assessed as recommended by the Consolidated Standards of Reporting Trials (CONSORT) guidelines [32]. Randomisation will be coordinated by the trial supervisor (CD), who will have no involvement in eligibility screening, enrolment or treatment processes. On receipt of signed consent forms and baseline measures, participants will be given a unique code and randomised using an online randomisation database [33]. The computer-generated randomisation schedule will apply a permuted block design to ensure that the groups are balanced periodically. The block size will be concealed until after the primary endpoint has been analysed. The randomisation list will remain with the trial supervisor for the full duration of the study. The list will be stored in an encrypted file on a password-protected computer in the trial supervisor’s office to ensure concealment of allocation. Allocation of participants will be communicated to administrative staff in the pain clinic by the trial supervisor via email. The administrative staff will store this allocation list in an encrypted file, on a password-protected computer in the pain clinic administrative office. Participants will be informed of their group allocation in writing by the administrative staff, who will send notification in sealed, opaque envelopes.

### Ethics

Ethical approval for the study has been granted by the Mater Misericordiae University Hospital Institutional Review Board (Ref No. 1/378/1864) and ethical exemption has been accepted by the UCD Human Research Ethics Committee – Sciences (Ref No. LS-E-17-03-Casey-Doody). The trial will be performed in accordance with the Declaration of Helsinki [34]. No significant adverse events are anticipated during this trial, but will be monitored and any adverse events that occur will be recorded and reported.

This protocol has been written in accordance with the Standard Protocol Items: Recommendations for Interventional Trials (SPIRIT) Statement [35] (See Additional file 1: ExACT Trial SPIRIT 2013 Checklist). Any significant modifications to the protocol will require a formal protocol amendment, agreed on by the project team and approved by the MMUH Institutional Review Board. Minor administrative changes to the protocol will be documented in a memorandum.

### Data collection methods

Data will be collected via self-report questionnaires at baseline (t_0_), post intervention (t_1_) and at 12-week follow-up (t_2_). Physical activity patterns will be measured objectively using Fitbit Zip™ (available from: http://www.fitbit.com) activity trackers and recorded from 1 week prior to commencing treatment through to completion of the intervention.

#### Baseline

A booklet of self-report questionnaires will be sent to participants via post, in advance of a scheduled face-to-face appointment with the lead researcher. This appointment will take place prior to randomisation and no more than 4 weeks prior to starting the intervention. Participants will be requested to complete the questionnaires at home and return them in person on the day of the appointment, when any questions related to the questionnaires can be clarified. The questionnaires will be checked for missing data by the lead researcher and completed by the participant where possible. The questionnaires will later be given to the trial supervisor who will de-identify and code them. No information about treatment allocation will be included on the questionnaires and they will be returned to the lead researcher who will enter the data in a secure, web-enabled information management system. One week of baseline physical activity data (daily step count, active minutes and distance travelled) will be collected prior to starting the interventions. The Fitbit Zip^TM^ activity tracker will be provided to participants at the baseline appointment, with a request to begin wearing the device at least 1 week prior to commencement of the intervention and to continue wearing it for the full duration of the 8-week intervention. An individual Fitbit account will be created for each participant using a unique email address, purposefully created for the study. Data for each participant will be retrieved remotely by the lead researcher via the Fitbit website.

#### Post intervention follow-up

The treating physiotherapist will provide follow-up questionnaires to all trial participants on the last day of the interventions. The participants will be provided with a private space to complete the questionnaires at the end of the last day and the physiotherapist will not be present with the participants whilst they fill in the questionnaires. The questionnaires will be placed in an opaque envelope and sealed before sending to the trial supervisor by registered post. The trial supervisor will check the questionnaires for missing data and will follow-up by post or with telephone calls to participants where possible. The questionnaires will be de-identified as outlined, before returning to the lead researcher who will enter the data into the electronic database.

#### Twelve-week follow-up

The 12-week follow-up time point has been selected as the primary endpoint for establishing effectiveness of the trial intervention. Participants will be contacted via telephone by the administrative staff in the pain clinic and questionnaires will be administered via post with an enclosed stamped addressed envelope for return to the trial supervisor (CD). The trial supervisor will follow up on any missing data again by telephone and a follow-up letter will be sent to participants who fail to return the questionnaires within 2 weeks. A final reminder telephone call will be made by the administrative staff in the pain clinic to participants who have not returned questionnaires after a further week.

### Blinding

Blinding of patients or the treating health professionals will not be possible due to the nature of the interventions. However, a position of clinical equipoise will be maintained, with patients advised verbally and in the Patient Information Leaflet, that they are being offered one of two treatments that are believed to be helpful for chronic pain but it is not known if one treatment is superior to the other. The lead researcher (MBC) will be blinded to group allocation when entering and analysing the data and the statistician (RS) analysing data will also be blinded. Methods to ensure maintenance of this blinding will include de-identification and coding of questionnaires by the trial supervisor (CD), who will be the only person to have access to the locked codes used for treatment allocation. The trial supervisor will also be responsible for randomisation and follow-up of missing data post intervention and at 12-week follow-up. Day-to-day communication and management of scheduling after randomisation will be coordinated by the administrative staff in the hospital pain clinic. Un-blinding of trial participants will occur only after creation of a final locked analysis dataset when the last patient has provided data at 12-week follow-up.

### Outcomes

The outcome measures included in this trial are based on the IMMPACT recommendations for outcome measures for use in chronic pain clinical trials [36]. A recent systematic review [22] recommended formally defining outcome measures as primary, secondary and treatment process measures in future RCTs featuring ACT.

#### Demographic data

Data collected will include age, gender, education level, relationship status and work status. Details regarding pain history will be collected including diagnosis (if applicable), and duration of pain.

##### Primary outcome

Pain interference at 12-week follow-up has been chosen as the primary outcome based on the IMMPACT recommendations and also a systematic review of ACT for chronic pain, which suggests using a measure of physical or social functioning, rather than pain or emotional functioning as a primary outcome [22]. The 12-week follow-up time point has been specified as the primary outcome as this has been suggested to present a low risk of bias in chronic pain trials [37]. Pain interference will be assessed with the Brief Pain Inventory-Interference Scale (BPIIS). This is a seven-item self-report questionnaire that measures the extent to which pain interferes with functions such as general activity, walking ability, normal work, mood, relations with people, enjoyment of life and sleep. The Brief Pain Inventory (BPI) has been shown to be a valid tool for assessing pain interference, with acceptable internal consistency [38]. Excellent test-retest reliability has been reported in a chronic pain cohort [39] and a reduction of 1 point on the interference scale has been recommended as a clinically meaningful change [40].

##### Secondary outcome measures

We hypothesise that participation in the interventions will influence many health dimensions and the following secondary outcomes will be assessed:

#### Pain intensity: Brief Pain Inventory (BPI) – Pain severity subscale

Pain intensity will be measured with the pain severity subscale of the BPI. Reductions of pain intensity of between 10 and 20% have been reported to represent a clinically meaningful change [40].

#### Patient Global Impression of Change (PGIC) scale

The PGIC scale will measure participants’ perceived level of improvement or lack thereof, due to the intervention. The PGIC has strong clinical relevance to the individual with good face and test-retest reliability [41]. The percentages of participants endorsing each of the responses will be reported as per the IMMPACT recommendations [40].

#### Patient satisfaction with treatment

This will be measured using a single question (question 7) from the Client Satisfaction Questionnaire-8 (CSQ-8), which is designed to measure client satisfaction with services [42]. The question will ask ‘In an overall, general sense, how satisfied are you with the service you have received?’ and four potential responses will be provided (very satisfied, mostly satisfied, indifferent or mildly dissatisfied and quite dissatisfied). The percentages of participants endorsing each of the responses will be reported.

#### Health-related quality of life: EuroQoL (EQ-5D-5 L)

The EQ-5D-5 L assesses quality of life in five dimensions: mobility, self-care, usual activities, pain/discomfort and anxiety/depression. Each dimension is scored out of a possible five levels of severity (no problems, slight problems, moderate problems, severe problems and extreme problems). The digits applied to each dimension are then combined in a five-digit number that describes the respondent’s health state. The EQ-5D-5 L has been shown to have good construct validity and responsiveness in a chronic pain cohort [43]. The EQ-5D-5 L will also be used to generate QALYs (Quality-adjusted Life Years), which will be required for a cost-consequence analysis.

#### Mood: Patient Health Questionnaire-9 (PHQ-9) and General Anxiety Disorder-7 (GAD-7)

Symptoms of depression will be assessed using the PHQ-9 [44], which is a nine-item questionnaire generating scores ranging from 0 to 27. A score of ≥ 10 is indicative of probable depressive disorder. The GAD-7 [45] assesses symptoms of anxiety experienced during the last two weeks. Both questionnaires are validated and commonly used to identify and measure symptoms of depression and anxiety in patients with chronic illness.

#### Health economics

A cost-consequence analysis will be performed and data related to costs and QALYs will be reported alongside outcomes. Patient healthcare resource utilisation data will be collected at baseline and at 12-week follow-up, using a self-report questionnaire that will record concomitant care (general practitioner and other healthcare professional contacts, emergency department visits and number of days of hospital in-patient stays related to pain management), investigations and pain interventions during the preceding 3-month period. The costs of providing the interventions will be calculated in terms of direct contact time with healthcare professionals and QALYs will be generated using the EQ-5D-5 L.

#### Medication

Current medications will be recorded at each time point with the BPI, which features a specific question related to medication usage. Any changes to medications will be reported.

#### Adverse events

The occurrence of any adverse events will be monitored by the treating clinicians throughout the 8-week intervention period. Any adverse events that occur will be recorded by the lead researcher and reported with the study results.

### Treatment process measures

#### Self-efficacy: Pain Self Efficacy Questionnaire (PSEQ)

Self-efficacy refers to a person’s confidence in their ability to perform activities despite pain [46] and has been identified as an important mediator in the relationship between pain and disability [47]. The PSEQ features ten items that produce a total score between 0 to 60, with higher scores indicating greater self- efficacy. Analyses have shown the PSEQ to have strong psychometric properties [48].

#### Pain catastrophising: Pain Catastrophising Scale (PCS)

Catastrophisation is defined as an elevated negative cognitive response to painful stimuli [49]. Change in pain catastrophising has been shown to mediate reductions in pain and disability [50]. The PCS consists of 13 items that refer to thoughts and feelings related to pain. Respondents are asked to rate the degree to which they experience each item on a 5-point scale 0 (not and all) to 4 (all the time) and items are summed to give a potential total score of 52. There are also three subscales within the PCS; rumination, magnification and helplessness. The PCS has been shown to have adequate to excellent internal consistency and validity [49, 51].

#### Fear avoidance: Tampa Scale of Kinesiophobia (TSK)

Fear avoidance beliefs have been shown to be associated with higher levels of disability [52] and worse prognosis in patients with low back pain [53]. The TSK is a 17-item questionnaire, which assesses fear of movement and re-injury. It has been reported to be a reliable and valid measure of fear of movement in individuals with chronic pain [54].

#### Chronic Pain Acceptance Questionnaire – 8 (CPAQ-8) and Committed Action Questionnaire – 8 (CAQ-8)

Pain acceptance and committed action are components of the ACT model and may be potential mediators of change, related to ACT. The CPAQ-8 is a shortened version of the original 20-item CPAQ, with two subscales; activity engagement and pain willingness. Each item is scored from 0 (never true) to 6 (always true). The CPAQ has been shown to be valid and reliable, with good internal consistency and sensitivity to change [55] and the shortened version has demonstrated a sound factor structure and similar psychometric properties to the CPAQ [56, 57]. The CAQ-8 is a shortened version of the original 18-item Committed Action Questionnaire, which assesses an individual’s persistence and flexibility in acting in the direction of valued goals [58]. The items are rated from 0 (never true) to 6 (always true). The CAQ-8 has shown comparable reliability and validity to the original version [59].

### Physical activity outcomes

Physical activity will be measured using a Fitbit Zip^TM^ wearable activity tracker. The trackers provide an objective indicator of physical activity behaviour and avoid common sources of error in subjective measurement (e.g. self-report measurement). The Fitbit Zip^TM^ has an internal memory that can store data for up to 30 days and data can be transferred wirelessly to the Fitbit website via a smartphone application or by computer using the dongle provided. The ease of download of information from the Fitbit website will enable the data to be captured remotely.

Data collected will include average weekly step count, distance travelled and active minutes. Participants will be provided with the activity tracker at the baseline appointment and instructed in how to use it. The activity trackers will be worn by participants for 1 week prior to starting the interventions and for the full duration of the interventions (8 weeks in total). The main time points for analysis will be the baseline week and the last week of the intervention.

The Fitbit Zip^TM^ has been found to be a valid measure of free-living physical activity in healthy adults [60]. A recent study comparing the reliability and validity of ten consumer activity trackers reported excellent test-retest reliability of the Fitbit Zip^TM^, which was also reported to be the most valid of the ten trackers [61]. To our knowledge this will be the first RCT to collect physical activity data related to the effect of a combined ACT and exercise intervention on physical activity patterns measured objectively in a chronic pain cohort.

### Sample size

Sample size was estimated using a target power of 80%, at a type I error rate of 0.05 and was calculated relative to the primary outcome measure; Pain severity subscale of the BPI. The statistical test assumed was an independent samples *t* test for group differences in the change from baseline to subsequent assessment, assuming that the randomisation ensures no systematic baseline or other covariate group differences. The minimal clinically significant difference for the interference scale of the BPI is 1 unit (standard deviation of improvement of 2 units) [40].

Calculation produced a suggested sample size of 64 per group. Allowing for potential attrition rate of 20% our final sample size is 80 participants per group.

### Statistical analysis

Outcome analyses will be conducted by a professional academic statistician (RS) who will be blinded to treatment group allocation. A statistical analysis plan (SAP) will be drafted outlining the precise model to be applied and finalised before the last patient assessment is completed. No interim analyses will be conducted. Descriptive statistics will be calculated for all outcome measures at each time point, including for continuous variables: means, standard deviations, or medians with ranges of scores; and for categorical variables: frequencies and percentages.

#### Analyses of effectiveness of primary and secondary outcomes

Descriptive and inferential statistics will be obtained using the appropriate statistical methods that seek to address our identified objectives. The primary analysis will compare the effect of the interventions on the primary outcome; pain interference at 12 weeks post completion of the intervention. All outcome analyses will be conducted according to an intention-to-treat principle, i.e. all randomised participants will be included in the main analysis and will be analysed as randomised, regardless of protocol adherence. Secondary analysis will include the analysis of the primary outcome post intervention and analysis of the secondary and treatment process outcomes detailed previously post intervention and at 12-week follow-up. Linear mixed models on the outcome measures over time will be fitted to evaluate the effectiveness of both interventions, which intrinsically adjusts for pre-treatment scores. Statistical significance will be assessed from a *p* value < 0.05 from the group by time interaction term. For all tests, two-sided *p* values will be used, which will be reported to four decimal places with *p* values < 0.001 reported as *p* < 0.001. The Bonferroni method will be used to appropriately adjust the overall level of significance for multiple secondary outcomes as applicable. In the case of a significant result, planned contrasts of the group effects at post treatment and at 12-week follow-up will be used to investigate the direction and pattern of effects, and outlined in advance in the SAP. As a key component of the reporting of the analyses of outcomes, the mean changes (irrespective of statistical significance) and correlations of the measures between the assessment time periods will be obtained. An up-to-date version of SPSS will be used to conduct the analyses.

#### Missing data

Careful attention will be paid to ensure that all participants are fully assessed at all time points. Baseline data will be checked for missing data by the lead researcher at the baseline assessment and participants will be encouraged to complete any missing answers. It is hoped that this process will help minimise missing data at follow-up as the senior researcher will be able to clarify any ambiguous questions face-to-face at baseline. However, the trial supervisor will follow up on any missing data by telephone or post at the subsequent time points. For the purpose of secondary analysis and as a sensitivity analysis for the primary outcome, multiple imputation will be considered for any measure with over 5% missing data, using a chained equations method robust to non-normally distributed data. This will be fully reported in line with the updated CONSORT recommendations [32]. All primary and secondary outcomes (excluding physical activity data), treatment process measures and pre-selected baseline covariates (age, gender, work and educational status, pain-intensity, anxiety, depression, self-efficacy and catastrophising) will be included in the imputation model. These baseline covariates have been selected based on studies that have examined predictors of outcome of multidisciplinary treatment in chronic pain [62, 63] .

#### Sensitivity analysis

The following sensitivity analyses will be undertaken and reported:

1. *A per-protocol analysis*: the per-protocol analysis will exclude participants found to be ineligible after randomisation and those who attend less than 50 % of the intervention. Both intention-to-treat and per-protocol analysis sets will be reported and superiority will be determined only if demonstrated with the primary intention to treat analysis
2. *Multiple imputation of missing data*: the results from a complete case analysis will be compared to those from imputed data to assess whether they change the interpretation of findings

#### Analysis of physical activity data

The following data will be collected for each trial participant at baseline and on completion of the treatment: average daily step count, distance travelled and active minutes. Only those participants who have worn the device for at least 4 out of 7 days during the baseline and final week will be included in the analyses. The number of participants reaching the global recommendations for physical activity for health [64] will also be recorded. Descriptive statistics will be obtained for the Fitbit variables at baseline and by treatment arm. Linear mixed models will be used to analyse the change in measures between groups.

### Methodology for the embedded qualitative study

#### Participants

Embedded qualitative interviews within this RCT will assist with interpretation of the findings of the study. Focus groups and individual, semi-structured, face-to-face interviews will be conducted with a purposeful sample of participants from both study arms, after the 12-week follow-up time point. Focus groups will be conducted initially and it is anticipated that ongoing analysis of focus group data may stimulate further research questions, which may be more appropriately investigated through individual interviews. People of different genders and ages and with different levels of pain intensity, pain interference, pain acceptance, fear avoidance, depression and anxiety will be invited to attend the focus groups. Depending on the trial progress, it may be useful to purposefully sample and interview a range of individual participants; for example, participants who have dropped out of the programme, participants who have not responded to the programme, participants who have responded well to the programme etc. to further explore and understand the reasons for same. These particular topics would be best investigated in a one-to-one interview. Selected participants will be sent a postal invitation to take part in the qualitative study by the pain clinic administrative staff and a copy of the trial Patient Information Leaflet will be included with the letter. The participants will be asked to respond by telephone or email, confirming whether or not they would like to take part. Those who opt to participate will be sent an appointment to attend either a focus group or individual interview. Travel expenses will be provided to participants attending from outside the local area.

#### Data collection

The qualitative data will be collected after the 12-week follow-up time point, and no longer than 6 months post completion of the intervention. The focus groups and individual interviews will be conducted by the lead researcher (MBC), trial supervisor (CD) or an external research assistant. The interviews will be semi-structured using a topic guide but participants will be encouraged to speak openly and freely about their experiences, positive and negative in relation to the interventions. Interviews will be scheduled for up to 90 min. The number of participants invited to the focus groups and individual interviews will be determined by ongoing data analysis and theme saturation.

#### Analysis

The focus group and individual interview audiotapes will be transcribed verbatim by a member of the project team, omitting any names, locations or information that could identify any individual. The de-identified transcripts will be analysed using an interpretative phenomenological approach [65]. Interpretative phenomenological analysis (IPA) is a qualitative analytic approach, commonly used in the field of health psychology [66]. The aim of IPA is to examine how people make sense of lived experiences. The approach is most often concerned with events that have significance to an individual, such as a major life experience, which would prompt a considerable amount of thinking and feeling as a person reflects on its meaning [65]. This study features an open research question, focussed on peoples’ experiences and views of the featured interventions for chronic pain. This type of research question is well suited to an IPA method, which aims to both give voice to the opinions of participants and to make sense of them by offering an interpretation [67]. Standards of verification will be adhered to including member checks, peer debriefing, external audit, negative case analysis, rich description including citations from the interview transcripts identified to participant and line number [68].

### Data management – data entry, coding, security, storage

In order to ensure patient confidentiality, all questionnaire data will be de-identified after collection and referred to only by a unique code assigned by the trial supervisor (CD). The trial supervisor will be the only person to hold the ‘key’ to re-identify the data for the full duration of the trial. The researchers who will have access to the de-identified datasets via the secure online database website are MBC, CD, KS and RS for the purpose of data entry (MBC), checking (CD and KS) and analysis (RS). All files will be encrypted and accessed via password-protected computers. Hard copies of the de-identified questionnaires will be stored in a locked cabinet in an office in the MMUH Pain Clinic.

Electronic data collected from Fitbit Zip^TM^ activity tracker will be stored on the Fitbit website and accessed only by the lead researcher (MBC) and trial supervisor (CD). Anonymised transcripts of the qualitative interviews will be accessed by the lead researcher and trial supervisor. Audio files of the interviews will be accessed by the lead researcher (MBC) who will transcribe and de-identify the data. The audio files will be destroyed once they have been transcribed. Audio files of the ACT sessions that are recorded for the purpose of fidelity assessment will be password protected and accessed only by the psychologists (DL and NL). These files will also be destroyed on completion of the fidelity assessment.

### Dissemination

The results of this trial will be published in peer-reviewed journals and will be disseminated at relevant conferences.

## Discussion

This paper describes the protocol of the ExACT trial, a RCT comparing the effectiveness of exercise combined with ACT, to a supervised exercise programme in reducing pain interference in a heterogeneous adult patient population with chronic pain. We have endeavoured to address recommendations that have been made to enhance the quality of research in the field of ACT, including choice of outcome measures and comparison to an active control group [22]. The inclusion of objective measurement of physical activity, using wearable activity trackers is novel in this field of research and highly relevant, considering the prevalence of co-morbidities in chronic pain patients. The inclusion criteria for the study are broad, in recognition that chronic pain is a heterogeneous condition, and with the aim of maximising the generalisability of the findings. Through the embedded qualitative study, we aim to provide insight into patients’ experiences and views of the interventions.

There are a number of limitations inherent in this trial of what have been termed ‘complex interventions’ [69, 70], most prominently concerning the inability to blind study participants and clinicians to treatment allocation. We have attempted to address issues of additional biases as far as has been practically possible through the provision of randomisation and concealment of allocation, strategies to minimise and manage incomplete outcome data, assessment of intervention fidelity, an adequate sample size with appropriate duration of follow-up and a priori specification of all primary and secondary outcomes as detailed in this study protocol.

To our knowledge, this will be the first RCT to assess the effectiveness of a combined Exercise and ACT intervention for chronic pain. The study results will add to current knowledge in the field of chronic pain management and will have the potential to inform the delivery of effective treatments for patients.

### Trial status

This trial is currently recruiting participants. It is anticipated that recruitment will be ongoing until the end of 2018.

## Additional file

Additional file 1: ExACT Trial SPIRIT 2013 Checklist. (DOC 122 kb)

## Abbreviations

ACT: Acceptance and Commitment Therapy
BPI: Brief Pain Inventory
CAQ: Committed Action Questionnaire
CPAQ: Chronic Pain Acceptance Questionnaire
EQ-5D-5 L: EuroQoL 5D-5 L
ExACT: Exercise and Acceptance and Commitment Therapy
IPA: Interpretative phenomenological analysis
PCS: Pain Catastrophising Scale
PSEQ: Pain Self Efficacy Questionnaire
QALY: Quality-adjusted Life Year
RCT: Randomised controlled trial
TSK: Tampa Scale for Kinesiophobia
